# Effectiveness of Cardiac Rehabilitation Services in Low- and Middle-Income Countries: A Systematic Review

**DOI:** 10.7759/cureus.50953

**Published:** 2023-12-22

**Authors:** Lilian Mbau, Prabhakar Mallya Prabhakar, Zahid Khan

**Affiliations:** 1 Cardiology, Kenya Cardiac Society, Nairobi, KEN; 2 Diabetologist and Physician, Prabhath Diabetes Care Centre, Udupi, IND; 3 Acute Medicine, Mid and South Essex NHS Foundation Trust, Southend on Sea, GBR; 4 Cardiology, Bart’s Heart Centre, London, GBR; 5 Cardiology and General Medicine, Barking, Havering and Redbridge University Hospitals NHS Trust, London, GBR; 6 Cardiology, Royal Free Hospital, London, GBR

**Keywords:** cardiac tele-rehabilitation, american heart association (aha) and american college of cardiology (acc), coronary artery disease (cad), quality of life (qol), cardiac rehabilitation (cr)

## Abstract

Cardiac rehabilitation (CR) is a cost-effective intervention that can reduce cardiovascular disease (CVD) morbidity and mortality by 20%. Despite the clear benefits of CR, it remains unavailable and underutilized. This study aimed to assess the effectiveness of different CR models in reducing CVD-related morbidity and mortality in low-and middle-income countries.

We conducted a systematic review of studies conducted in low- and middle-income countries that assessed at least one of the three phases of CR (inpatient rehabilitation, outpatient rehabilitation in a hospital, or community setting and maintenance). The primary outcomes of interest were mortality (all-cause and CVD-specific), CVD-related morbidity, functional capacity, risk factor reduction, and quality of life (QoL).

The electronic search retrieved 1,102 studies, of which 22 were retrieved and included in the review. These studies were conducted between 2011 and 2022 and the majority (18) were conducted in Asia. All studies except one were randomized controlled trials (RCTs), and all except one were conducted at a single site. The target population in most studies (16) included patients with coronary artery disease (CAD). Seven studies have incorporated digital technology. Only one study has reported a significant reduction in all-cause mortality. Thirteen studies reported data on functional capacity, and 16 on quality of life (QoL), showing statistically significant improvements. Data on risk factors, anxiety, and depression have shown mixed results. CR is effective in low- and middle-income countries, and strategies to scale it up using locally available resources tailored to the patient population should be adopted.

## Introduction and background

Cardiac rehabilitation (CR) refers to programs that provide education, exercise, and behavioral modification to improve the quality of life (QoL) of patients with cardiovascular diseases (CVDs). The primary goal is to optimize the patient’s physical, social, psychological, and vocational functions through lifestyle modifications and physical exercise [1]. The components of CR include prescribing exercise within safe limits and interventions to reduce CVD risk factors, such as smoking cessation, lipid-lowering, weight loss, consumption of healthy foods, blood sugar control, blood pressure (BP) control, and increasing physical activity. It also focuses on improving psychological well-being, which is critical for reducing risks [1].

CR programs are typically delivered in three phases. The initial phase is inpatient rehabilitation, which begins during admission and involves ambulation in the ward using equipment, such as stationary bikes or treadmills. The patient was referred to the next phase of outpatient rehabilitation in a hospital setting. It is physician-supervised and conducted by a multidisciplinary team for up to 36 sessions (usually thrice weekly). However, growing evidence shows that this phase can be converted to home-based CR, especially for patients who have difficulty accessing health facilities. The final step is maintenance, in which the patient independently continues with physical activity and risk factor modification without cardiac monitoring [2]. CR should focus on patients' nutritional counseling, weight management, lipid management, diabetes control, smoking cessation, psycho-social management, and exercise or physical activity counseling. The main indications for CR include acute coronary syndrome, stable angina, congestive heart failure, post-coronary artery bypass grafting, cardiac transplantation, post-percutaneous intervention, and valvular surgery. Contraindications to CR include acute decompensated heart failure, severe pulmonary hypertension, unstable angina, intracavitary thrombus, recent thrombophlebitis with or without pulmonary embolism, severe obstructive cardiomyopathy, severe aortic stenosis, musculoskeletal conditions preventing adequate patient participation in exercise and uncontrolled infectious or inflammatory disease.

CR is cost-effective and can reduce CVD morbidity and mortality by 20% [3,4]. It reduces cardiovascular risk factors and improves adherence to medications, QoL, and functional status [1,5]. Guidelines by the American Heart Association (AHA) and American College of Cardiology (ACC) give a class 1 recommendation for referral to CR services for patients with myocardial infarction, coronary artery bypass graft, percutaneous coronary intervention, chronic stable angina, heart failure, peripheral arterial disease, and CVD prevention in women [1]. Despite the clear benefits of CR, it remains unavailable and underutilized [2]. Only 38.8% of countries globally have CR programs, with the lowest in low- and middle-income countries (LMIC) [6]. A study conducted in the US in 2015 reported referral rates of 62%, with only 23% attending at least one session and only 5% completing 36 or more sessions [7].

Data on barriers to under-utilization of CR are primarily from high-income and middle-income countries. Some of the strategies proposed to increase CR utilization, especially in LMIC, include the development of public health policies to promote and prioritize resources for CR, integration of CR within mainstream cardiology services, incorporating pre-service CR training for health providers, task-shifting elements of CR to lower cadres such as nurses, increasing flexibility of programs, and decentralization programs [6]. Referrals can also be improved by creating flexible schedules, educating caregivers, mobile-based and home-based exercise programs, and lowering costs [2]. The proposed models for the delivery of CR services in resource-limited settings include home-based programs, Internet-based telehealth, and mobile phones to deliver CR. These models can reduce costs and offer flexibility. Unfortunately, evidence of the effectiveness of these models in these settings remains limited [8].

Aims and objectives

This study aimed to assess the effectiveness of different CR models in reducing CVD-related morbidity and mortality in limited-resource settings. CR has clear benefits related to CVD risk factor reduction, improved adherence, and reduced CVD-related morbidity and mortality [1,5]. Despite these benefits, it remains unavailable and underutilized, especially in limited-resource settings. The barriers to CR provision in these settings are fully understood [6]. The delivery of CR programs is influenced by the resources available. Strategies have been proposed to improve the utilization of CR services in poor resource settings. It is therefore vital to establish the effectiveness of these strategies. This information will go a long way to support advocacy efforts to increase access and utilization of CR services in resource-limited settings.

## Review

Methods

Types of Studies

Randomized controlled trials (RCTs) were included, as well as other studies such as cross-sectional studies, cohort studies and longitudinal studies because the preliminary literature review suggests that the pool of studies and reports on the proposed topic may be limited. Studies were included irrespective of the date of publication. Studies in other languages than English were excluded.

Types of Participants and Settings

Participants included patients with CVDs participating in CR services at any of the three phases in LMICs. CVDs included heart failure, myocardial infarction, angina, cardiomyopathy, pacemakers, heart valve replacement, concomitant pulmonary disease and cardiac transplant. LMICs are defined by The World Bank Group [9]. Adult patients, both males and females were included. Studies that have only a subset of participants were also included.

Types of Interventions

Studies assessing at least one of the three phases of CR (inpatient rehabilitation, outpatient rehabilitation in a hospital or community setting and maintenance) were included.

Types of Outcome Measures

The primary outcomes include all-cause mortality (total deaths), CVD-related mortality (deaths related to the CVD that led to the referral for the CR services), CVD-related morbidity (incidence and hospitalisation of recurrent episode and complications), functional capacity, risk factor reduction (lipid-lowering, smoking cessation, weight loss, BP control, blood sugar control, consumption of healthy diets, increased physical activity) and QoL (psychological and physical well-being). The secondary outcome is cost-effectiveness.

Literature Search 

The following databases were searched for primary studies: MEDLINE, Embase, Cochrane Central Register of Controlled Trials. Trial Registries searched include the US National Institutes of Health (NIH) and the World Health Organization International Clinical Trials Registry Platform (WHOICTR). Studies from reference lists of systematic reviews from the Cochrane Database of Systematic Reviews (CDSR) were also included.

A clear and focused research question was determined. The search question was translated into key terms according to relevant elements from PICOS: Patient population, intervention, comparator, outcome and study design. The comparator and outcome elements were omitted as they usually do not appear in the indexing, abstract or title [10]. Database-specific subject headings and index terms (MESH in MEDLINE) were referenced [10]. Database-appropriate syntax, with parentheses, Boolean operators (AND, OR), and field codes were applied [11]. Synonyms and variations in the search terms (spelling differences, abbreviation and truncations) were identified.

The key terms used were cardiac rehabilitation, cardiopulmonary rehabilitation, cardiovascular rehabilitation, CVDs, heart failure, chronic heart failure (CHF), myocardial ischemia, myocardial infarction, angina pectoris, angina, pectoris, anginas, cardiomyopathy, cardiomyopathies, pacemaker, artificial. The index term used was “cardiac rehabilitation.” The final search strategy used key terms and index terms identified. The search strategy was tested for completeness in MEDLINE and after optimization translated to the other databases. Databases were searched from inception to date.

Data Collection and Analysis

**Figure 1:**
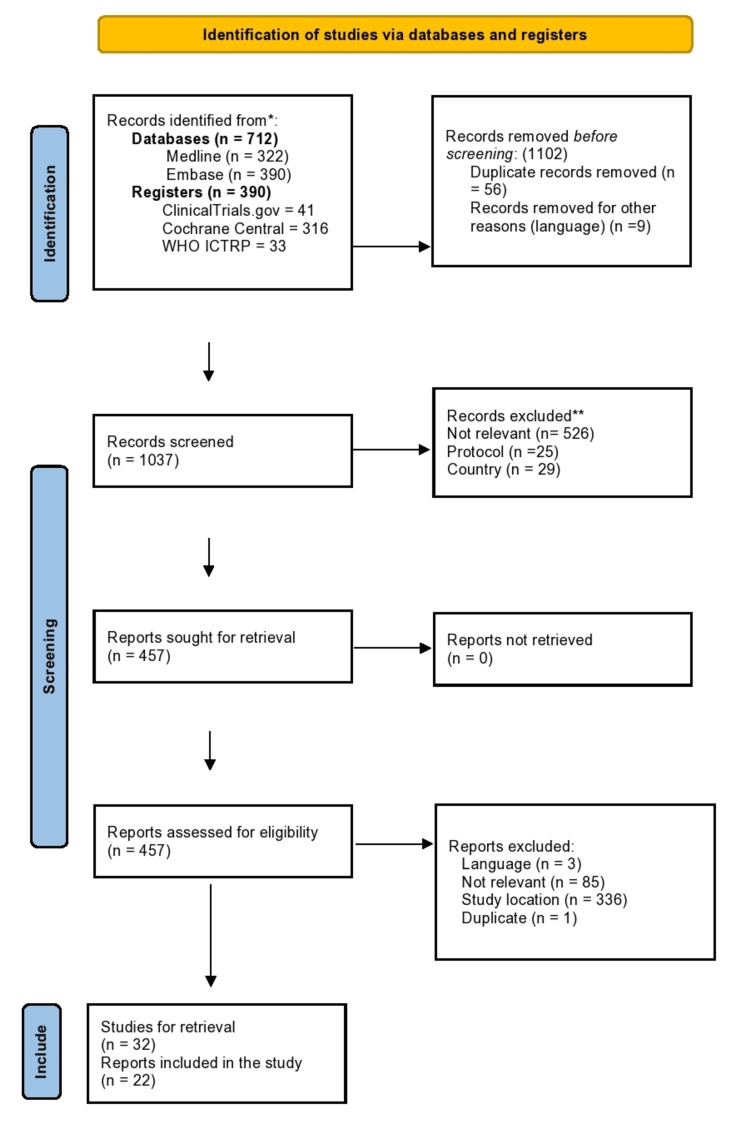
PRISMA 2020 flow diagram for new systematic reviews, which included searches of databases, registers and other sources PRISMA - Preferred Reporting Items for Systematic Reviews and Meta-Analyses [12]

Titles and abstracts of records were identified and screened for eligibility. Thereafter, the full-text reports were screened to identify the studies to be included. The studies excluded have been listed in a table. The selection process has been summarized in the Preferred Reporting Items for Systematic Reviews and a Meta-Analysis (PRISMA) flow diagram (Figure 1) [12].

Data Extraction and Management

A data extraction matrix was used to extract the data from the reports. Extraction was done by the principal investigator and included data on the relevant inclusion criteria (study design, study population, intervention, duration of the study) and risk of bias. The following study characteristics were extracted; methods (location, study setting, duration of the study, sample size and study design), participants (study population, CVD, age, sex), interventions (intervention, control), outcomes (primary and secondary outcomes).

Assessment of Risk Bias in Included Studies

The risk of bias was assessed for each study using the assessment tool modified by the EPOC Group [13]. A risk of bias table was created to document the source of bias as either high, low or unclear. An overall risk of bias assessment for each study was assigned as low risk if the risk of bias was low in all key domains; unclear risk if it was unclear in at least one domain or the risk can raise doubts about the conclusion and high risk if the risk was high in at least one domain or serious bias that can affect conclusions.

Quality Assessment

The quality of randomized and non-randomized control trials was assessed using the revised Cochrane risk of bias tools for randomized trials (ROB-2) [14]. The quality of other quantitative studies was assessed based on the National Institute of Health (NIH) Quality Assessment Tool for Observational Cohort and Cross-Sectional Studies [15]. This form appraised the reliability, validity and generalizability of the quantitative studies. The NIH quality assessment tool uses 13 criteria to assess and rate the quality of studies. This included the research question, study population, sample size estimation, exposure and outcome assessment, loss to follow-up, and statistical analysis. General guidance is provided for determining the overall quality of the studies and to grade their level of quality as good, fair, or poor. The quality of the study was included in the “Summary of findings” table (s).

Risk of Bias in Included Studies

**Figure 2:**
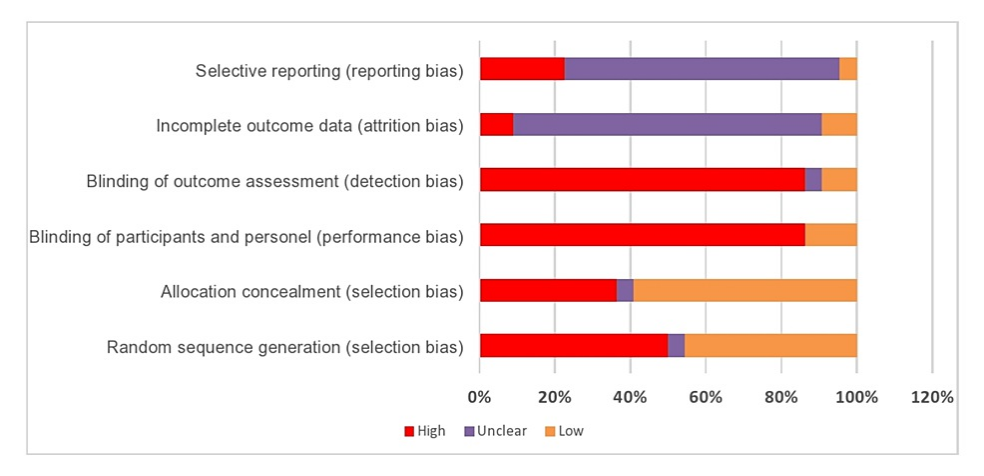
Review the author’s judgment about the bias presented as percentages across all included studies

**Figure 3:**
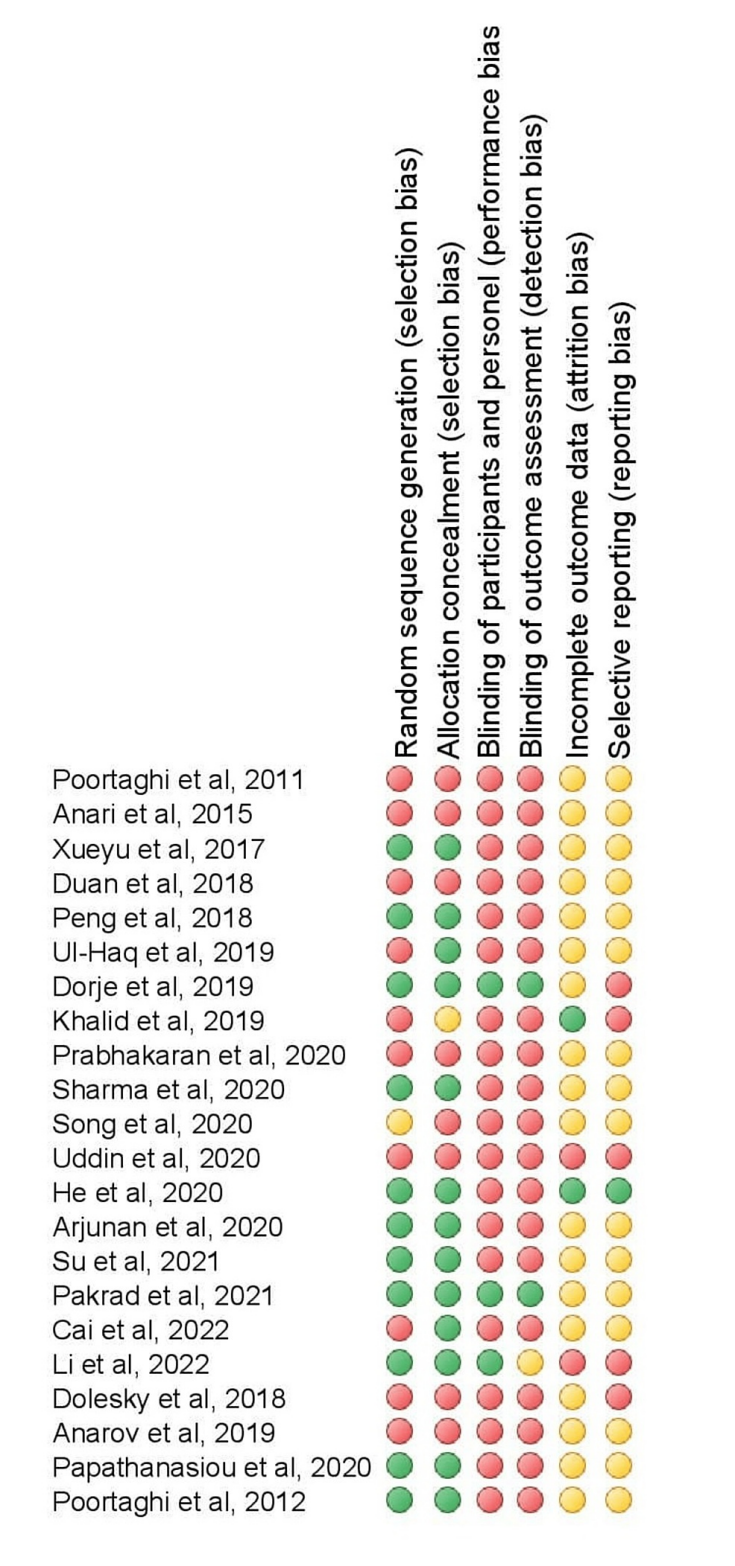
Author’s judgment about the bias presented as percentages across all included studies Study references: [16-37]

The overall risk of bias in the included studies was generally unclear or high. Most of the RCTs were single-blinded. Double blinding is difficult to achieve due to the nature of exercise interventions. This is summarized in Figures 2, 3.

Results

The electronic search retrieved a total of 1,102 studies. After removing duplicates and other ineligible studies, 1,037 records were eligible for screening. Following the screening of titles, 528 records did not meet the inclusion criteria, 25 were protocols, and 29 were from high-income countries (HIC). In total, 457 abstracts were retrieved and assessed for eligibility. Of these, we excluded 336 reports from HIC, 85 of which did not meet the inclusion criteria, and three of which were not available in English. Of the 32 remaining papers, 22 were available and were included in the study. The study selection process is summarized in a PRISMA flow diagram (Figure 1). A total of 22 studies fulfilled the inclusion criteria, and full papers were available for review. It is interesting to note that these studies were conducted within the last decade, between 2011 and 2022. The majority of the studies (18) were conducted in Asia [16-33], while three [34-36] were conducted in Europe and one in South America [37].

All studies were RCTs except one [17], which was a prospective interventional cohort study. The sample sizes across various studies varied from 36 [35] to 3,959 [24]. All the studies were conducted at a single site, except one that was conducted in 24 medical centers across India [24]. The study duration varied from wight weeks to three years. The target population in most studies (16/22) included patients with coronary artery disease (CAD) [16,17,19,21-28,30,31,33,35,37]. Five of the studies targeted patients with CHF [18,20,29,34,36] and one study recruited patients with atrial fibrillation (AF) [32]. The study setting was hospital-based, home-based, or a combination of both. Interventions with a home-based component were 16 [16,18-22,25,27-32,34,35,37], while those that were purely hospital-based were only five [17,23,24,33,36]. All interventions had an exercise component, while others involved a combination of exercise and education on CR and/or lifestyle modification. Interventions from seven studies [19,20,22,26,31,32] involved the use of digital technology (either mobile phones or web-based platforms).

The outcomes reported in these studies include functional capacity, cardiovascular risk factors (behavioral and physiological), QoL, and clinical outcomes (mortality and morbidity). The parameters commonly used to assess functional capacity were the 6-minute walk distance (6 MWD) and peak VO2. Various questionnaires were used to assess quality of life. Only three studies [24,28,37] reported the effect of interventions on clinical outcomes, morbidity, and mortality (all-cause and cardiovascular-related).

Demographic Characteristics 

In the majority of studies (20/22), the majority of study participants were male. The mean age of the study participants in both the control and intervention groups ranged from 46 to 78 years. The demographic characteristics of the populations in the studies included are summarized in Table 1.

**Table 1:** Study demographics. Randomized controlled trial (RCT), Coronary artery disease (CAD), Coronary artery bypass grafting (CABG), Major adverse CV events (MACE), Myocardial infarction in the absence of obstructive CAD (MINOCA), Percutaneous transluminal coronary angioplasty (PTCA), Myocardial infarction (MI), The New York Heart Association (NYHA), Chronic heart failure (CHF), Percutaneous Coronary Intervention (PCI), Left ventricular ejection fraction (LVEF), Atrial fibrillation (AF).

Author (Year of publication)	Country	Study design	Sample	Age, Mean±SD			Male sex, n (%)		Target population
				Intervention	Control		Intervention	Control	
Poortaghi et al., 2011 [16]	Iran	RCT	80	57.78±1.36	57.05±1.51		31 (77.5)	29 (72.5)	Patients’ post-CABG, MI or PTCA
Anari et al., 2015 [17]	Iran	Prospective interventional cohort study	108	58.25±9.83			86 (79.6)		Patients with a previous diagnosis of CAD (post CABG or PTCA)
Xueyu et al., 2017 [18]	China	RCT	78	76±4.39		78±3.26	24 (63)	30 (75)	Stable Class II or III CHF (NYHA) with limitations in PA and comfortable at rest but symptomatic with or less than ordinary PA, 70 or more years
Duan et al., 2018 [19]	China	RCT	114	51.57±11.57		45.80 ±14.68	48.1%	45.6%	Outpatient with CAD
Peng et al., 2018 [20]	China	RCT	98	66.3 (10.50)			30 (61.2)	28(57.1)	CHF for at least 3 months, NYHA 1 to III, more than 18yrs, clinically stable with regular medications for at least 4 weeks
Ul-Haq et al., 2019 [21]	Pakistan	RCT	206	53.6±8.3			69 (71.88)	81(81.82)	Patients with the first MI
Dorje et al., 2019 [22]	China	RCT	312	61.9±8.7		59.1±9.4	126(81)	128(82)	Patients 18 years and older with CAD post-PCI
Khalid et al., 2019 [23]	Pakistan	RCT	26	55.77±10.257		57.23±9.757	16 (61.5)		Stable post-MI patients, 35 years and above
Prabhakaran et al., 2020 [24]	India	RCT	3959	53.4±10.8		53.4±11.0	1709(85.9)	1699 (86.2)	Acute MI, 18-80 years, past 14 days
Sharma et al., 2020 [25]	India	RCT	66	51.51±8.15		53.15±11.59	31(93.9)	26(78.8)	30 - 65 years old, recent MI on conservative management without involving conservative procedures (PCI, CABG), 10 days to 2 months post-MI, left ventricular dysfunction (NYHA class I and II, LVEF 30-50%.
Song et al., 2020 [26]	China	RCT	106	54.83±9.13		54.17±8.76	40 (83.33)	43(89.60)	Patients with stable CAD
Uddin et al., 2020 [27]	Bangladesh	Quasi-RCT	142	55±6		54±6	63(89)	66(93)	Post-CABG
He et al., 2020 [28]	China	RCT	524	60.9±12.9		60.6±12.7	124 (47.7)	120 (45.8)	Patients with MINOCA
Arjunan et al., 2020 [29]	India	RCT	200	59.57±11.16			79(79)	78(78)	Patients admitted with CHF
Su et al., 2021 [30]	China	RCT	146	56.03±7.02		55.53±7.30	60(82.2)	62(84.9)	Initial diagnosis of CAD based on angiography or exacerbation of CAD in previously diagnosed
Pakrad et al., 2021 [31]	Iran	RCT	88	62.9±9.8		62.6±8.1	38(86.4(	36(81.8)	Post-CABG patients
Cai et al., 2022 [32]	China	RCT	100	57±9		57±11	32 (66.7)	31(63.3)	Patients with AF who underwent ablation.
Li et al., 2022 [33]	China	RCT	89	60.2 ±11.3			33 (79)	39 (83)	Patients 45 years and above were diagnosed with CAD by angiogram and received PCI
Doletsky et al., 2018 [34]	Russia	RCT	46	59.1±13.9		62.6±9.8	20(90.9)	22(91.7)	Decompensated HF (CHF with moderate or severe symptoms - class III or IV NYHA)
Aranov et al., 2019 [35]	Russia	RCT	36				18(100)	18 (100)	Male patients 3-8 weeks after CABG
Papathanasiou et al., 2020 [36]	Bulgaria	RCT	120	63.82±6.71		63.65±6.71	35 (58.33)	35(58.33)	Stable CHF, NYHA classes II to IIIB, LVEF equal or more than 40, clinically and pharmacologically stable for more than 3 months
Chaves et al., 2019 [37]	Brazil	RCT	115	59.5±9.4			82(71.3)		Patients post- CABG, myocardial infarction (MI) or percutaneous transluminal coronary angioplasty (PTCA)

Effect of the interventions

Mortality

Only one study has reported data on all-cause and CVD-related mortality [28]. The intervention included both hospital- and home-based targeted patients with myocardial infarction in the absence of obstructive CAD (MINOCA). Exercise was provided under the supervision of a physician in the hospital three times a week for 20-30 minutes on a treadmill or bicycle at 65%-75% of the symptom-limited maximal heart rate. Thereafter, home-based training was carried out three times a week (52 sessions per year), involving moderate continuous training at moderate intensity (65%-75% peak heart rate) for 37 min. After three years, all-cause mortality occurred in 60 individuals with a significant reduction in the intervention group (p<0.05), while 136 individuals experienced major adverse CV events (MACE) with a significant reduction in the intervention group (p<0.01).

Morbidity

Five studies reported data on cardiovascular-related morbidity [30-32,35,37]. One study lacked the statistical power to provide evidence of differences in MACE [24]. Pakrad et al. [31] deployed a hospital- and home-based intervention involving a one-month supervised CR in a hospital and three months of remote CR using a mobile application. recruited patients who had undergone CABG. Communication with the patient was performed biweekly by a nurse at months 1 to 4 to control risk factors. The control only received one-month supervised CR in the hospital. At the final assessment at four months, there were four hospitalizations in the control group and none in the intervention group (two of the hospitalizations were non-cardiac (p=0.049). Cai et al. [32] implemented a home-based patient-tailored and mobile application guided CR targeting patients with AF who had undergone an ablation. The control group underwent a standard 12-week CR. This study reported no significant differences in AF recurrence after ablation between the control and intervention groups.

Aranov et al. [35] implemented a medical center and home-based intervention where patients who had undergone CABG engaged in 60 min exercise in a controlled setting (medical center) three times a week for four months followed by home-based exercises. At 12 months follow-up there were fewer CVD complications in the intervention group than in the control group (11.1% vs. 39.2%, respectively). Angina was reported in five (27.5%) patients in the control group and in none in the intervention group. All CV complications (angina, MI, revascularization, and CAD-related hospitalization) were three times less frequent in the study group. All patients in the intervention group returned to work within a year of enrolment compared to 82.4% in the control group. After 12 months of follow-up, 5.6% of the study group and 29.4% of the control group experienced worsening of clinical parameters (p<0.05). Patients in the study group were four times more likely to experience clinical improvement than those in the control group (p<0.05). Chaves et al. [37] conducted a study with three arms, comprehensive CR (CCR) who received exercise and education, exercise-only CR and wait-list control who received usual care where patients had follow-up appointments with a physician and no CR. The study recruited patients who underwent CABG, MI, or PTCA. CR was performed in the hospital and community. At one-year follow-up, nonfatal MI (p<0.04), percutaneous coronary interventions (p<0.03), and hospitalizations (p<0.03) were significantly lower in the CR group than in the control group.

Su et al. [30] compared a home-based nurse-led eHealth CR (NeCR) intervention with usual care among patients who had an initial diagnosis of CAD based on angiography or exacerbation of CAD in previously diagnosed patients. Two participants in the intervention group were rehospitalized for chest pain and heaviness, and one underwent PCI. In the control group, two patients reported emergency unit visits for cardiac-related symptoms (tachycardia and bradycardia) and two reported chest pain and were re-hospitalized for PCI. A statistically significant difference was observed between the groups. The time to admission was longer in the NeCR group, but the difference was not statistically significant.

Functional Capacity

A total of 13 studies reported functional capacity outcomes [18,20,22,23,25-27,31,32,34-37]. The commonly used measures of functional capacity were the 6-minute walk distance (6 MWD) and VO2 (maximal or peak oxygen consumption). Other measures included Metabolic Equivalents (METs), graded cycle ergometer test (CE-test), Timed Up and Go (TUG) test, m-Borgs perceived exertion scale (mBPES), and incremental shuttle walk test. All studies showed improvement in functional capacity. The results are summarized in Table 2.

**Table 2:** Summary of functional capacity outcome for the studies included. Metabolic Equivalents (METs), Six-minute walk distance (6 MWD), VO2 (maximal or peak oxygen consumption), Graded cycle ergometer test (CE-test), Timed Up and Go (TUG) test, m-Borgs perceived exertion scale (mBPES),

Author (year)	CVD	Settings	Intervention	Measure	Functional capacity
Xueyu et al., 2017 [18]	CHF	Hospital and home	Transitional care program - individual discharge plan prepared for management of CHF and low-intensity exercise (walking protocol).	6MWD, Timed Up and Go (TUG) test	Significant improvement in 6MWD and TUG
Peng et al., 2018 [20]	CHF	Home-based	Home-based Telehealth exercise training (brochure, exercise training, regular telephone and instant messaging follow-up and consultation)	6MWD	Significant improvement in 6MWD
Dorje et al., 2019 [22]	CAD	Home-based	2-month intense program and 4-month step-down phase of smartphone-based CR and secondary prevention program delivered via social media platform WeChat (SMART-CR/SP).	6MWD	Improvement in 6MWT was greater in the intervention group at 2 months and maintained at 6 months.
Khalid et al., 2019 [23]	CAD	Hospital-based	Supervised resistance interval training in addition to Aerobic Interval Training	6MWD, Peak VO_2_	Significant increase in peak VO_2_
Sharma et al., 2020 [25]	CAD	Hospital and home	Three supervised integrated approach of Yoga Therapy (IAYT) classes at the hospital yoga centre supervised by a yoga therapist/cardiologist.	Metabolic equivalents (METs)	Improved METs (p=0.0)
Song et al., 2020 [26]	CAD	Home-based	Smart-phone-based telemonitored cardiac rehabilitation.	VO_2 _peak	A significant difference in VO_2_ peak between the 2 groups
Uddin et al., 2020 [27]	CAD	Home-based	In-hospital CR class, locally developed educational booklet, telephone follow up	Maximal VO_2_	Significant increase (<0.05)
Pakrad et al., 2021 [31]	CAD	Hospital and home	Hybrid CR - 1 month supervised and an additional 3 months remotely. Patients were given an app and communicated biweekly with the nurse for 1- 4 months	METs	Significant 1.5 MET increase in the intervention group
Cai et al., 2022 [32]	AF	Home-based	Home-based patient-tailored and mobile application-guided cardiac telerehabilitation	Peak V0_2_	Both groups reported a significant increase
Doletsky et al., 2018 [34]	HF	Hospital and home	Moderate-intensity aerobic training (Interval training for 3 weeks, five days a week starting as an in-patient and continuing as an outpatient)	6MWD, peak VO_2_	VO2 peak increased significantly (p<0.001). 6MWT distance increased in the intervention group.
Aranov et al., 2019 [35]	CAD	Hospital and home	Exercise in a controlled setting (medical centre) followed by home-based exercise	Graded cycle ergometer test (CE-test)	Physical endurance increased by 32.6% (p<0.05) at 12 months
Papathanasiou et al., 2020 [36]	CHF	Hospital-based	Modified group High-intensity aerobic interval training (HIAIT)	6MWD, m-Borgs perceived exertion scale (mBPES), Peak VO_2_.	Significant improvement in 6MWT (P<001), and VO_2_ peak (<0.001). Significant decrease in mBPES in the intervention group (p<0.001).
Chaves et al., 2019 [37]	CAD	Hospital and home	There was comprehensive CR (CCR) - exercise and education and CR (exercise only). At the community, they were encouraged to undertake 30 or more minutes of physical activity at moderate to vigorous intensity at least 5 days per week	Incremental shuttles walk test, 7 metabolic equivalents target)	Improved functional capacity in controls electing exercise only CR after 6 months

Risk Factor Reduction

Thirteen studies reported the outcomes of risk factor reduction [17,19,22,24-27,29,30,32,33,35,37]. The commonly reported risk factors were body mass index (BMI), body weight, waist circumference (WC), waist-to-hip ratio, blood lipids (low-density lipoprotein (LDL), triglycerides (TG), High-Density Lipoprotein (HDL), and total cholesterol)), random blood sugar (RBS), systolic BP (SBP), diastolic BP (DBP), HBa1c, smoking cessation, physical activity levels, and knowledge.

Six studies reported data on BMI [19,22,27,29,30,35]. Of these studies, the majority (4/6) [19,22,29,30] reported no significant difference in BMI, while 2 studies demonstrated a significant difference in favor of the intervention group [27,35]. Studies that showed improved BMI had interventions delivered at both hospital and home settings, targeting patients with CAD. With regard to other physical measurements, two studies reported WC, with one demonstrating a significant decrease in favor of the intervention group [17] and the other with no significant difference [30]. With regard to the physiological risk factors, five studies examined blood lipids [22,25,26,29,35], BP [17,22,27,29,30], and four blood sugar [26,27,29,35]. One study showed improvements in all three parameters (BP, blood sugar, and blood lipids) [29]. This was a nurse-led CR program targeting patients with CHF, where structured teaching was offered on diet, disease, medication, exercise, smoking cessation, home care, lifestyle modification in the hospital, and a booklet on CR given at discharge. The patients received phone reminders every two weeks on CR practices.

Regarding behavioral risk factors, two studies reported data on smoking cessation [22,30] and one on physical activity [32]. Su et al. [30] reported a significant difference in favor of the intervention for smoking cessation while Dorje et al. reported no significant difference. The intervention by Su et al. was also nurse-led and involved the use of an eHealth platform after discharge, where knowledge was shared, and goal attainment was monitored. Cai et al. [32] conducted a home-based patient-tailored and mobile application-guided cardiac tele-rehabilitation intervention targeting patients with AF after ablation. The study reported significantly higher physical activity levels in the intervention group after 12 weeks (p = 0.002). These results are summarized in Table 3.

**Table 3:** Summary of outcomes on risk factor reduction in the studies included. Random blood sugar (RBS), systolic blood pressure (SBP), diastolic blood pressure (DBP), waist circumference (WC), Body mass index (BMI), Hemoglobin A1c (HbA1c), Integrated approach of Yoga Therapy (IAYT), Cardiac Rehabilitation (CR), LDL (Low density lipoprotein), Coronary heart disease (CHD), Smartphone and social media-based CR and secondary prevention in China (SMART-CR/SP), Yoga-based CR (Yoga-based CR), Triglycerides (TG), High density lipoprotein (HDL), Glycated haemoglobin (HbA1c), Random blood sugar (RBS), Nurse -led eHealth CR (NeCR), Blood pressure (BP),  Waist circumference (WC), International Physical Activity Questionnaires (IPAQ), comprehensive CR (CCR), Coronary artery disease (CAD).

Author (Year)	CVD	Settings	Intervention	Risk factor reduction
Anari et al., 2015 [17]	CAD	Hospital-based	Exercise training, dietary recommendations	Significant decrease in WC, resting SBP and DBP
Duan et al., 2018 [19]	CAD	Home-based	Web-based intervention - First 4 weeks of physical activity and subsequent 4 weeks of fruit and vegetable consumption	No significant difference in BMI
Dorje et al., 2019 [22]	CAD	Home-based	2-month intense program and 4-month step-down phase of smartphone-based CR and secondary prevention program delivered via social media platform WeChat (SMART-CR/SP).	Significantly higher knowledge of CHD, significantly lower SBP, lipid concentration (Total cholesterol and LDL). No significant difference in smoking cessation, BMI, waist-hip ratio
Prabhakaran et al., 2020 [24]	CAD	Hospital-based	Yoga-based CR (Yoga-CaRe) - 13 direct contact sessions spread over 12 weeks. The first 2 sessions were delivered individually and the rest in groups at the hospital.	No difference in tobacco cessation at 12 weeks
Sharma et al., 2020 [25]	CAD	Hospital and home	Three supervised integrated approach of Yoga Therapy (IAYT) classes 3 days per week for 12 weeks at the hospital yoga centre	No significant difference in lipid levels
Song et al., 2020 [26]	CAD	Home-based	Smart-phone-based telemonitored cardiac rehabilitation.	Significant difference in changes in exercise habits between the two groups (0.020). No difference in the 2 groups in control of blood lipids and blood glucose
Uddin et al., 2020 [27]	CAD	Home-based	In-hospital CR class, locally developed educational booklet, telephone follow up	Significant decrease in BMI (0.008), TL (0.001), LDL (0.001), TG <0.002, HDL <0,01). There was no significant difference in BP and Hba1c
Arjunan et al., 2020 [29]	CHF	Hospital and home	Nurse-led CR - structured teaching, booklet on CR (Healthy way to the healthy heart) given at discharge and fortnightly phone reminders about good CR practices.	Significant difference in DBP, RBS and HDL. No change in BMI.
Su et al., 2021 [30]	CAD	Home-based	NeCR (Nurse -led eHealth cardiac rehabilitation) - In-person session by the nurse to develop an individualised plan and after discharge, an eHealth platform was used to share knowledge and monitor goal attainment	No significant difference in BP, BMI, body weight, or WC. A significant difference in smoking cessation.
Cai et al., 2022 [32]	AF	Home-based	Home-based patient-tailored and mobile application-guided cardiac telerehabilitation	IPAQ survey - Physical activity levels were significantly higher in the intervention group after 12 weeks (p=0.002)
Li et al., 2022 [33]	CAD	Hospital	Micro-lecture education to cover CR and secondary prevention is given in the ward twice a day	The rate of re-smoking in intervention was significantly lower than in control. The frequency of exercise is better than control. Significantly higher increase in exercise duration in the intervention group (0.000).
Aranov et al., 2019 [35]	CAD	Hospital and home	Exercise in a controlled setting (medical centre) followed by home-based exercise	Stable blood total and LDL cholesterol in intervention parameters, increased levels in the control group by 10.2% and 15.6%. Mean (p<0.05). Mean BMI did not change in the study group but increased significantly in the control group by 4.4% (p<0.05). No significant difference in blood glucose levels
Chaves et al., 2019 [37]	CAD	Hospital and home	There was comprehensive CR (CCR) - exercise and education and CR (exercise only). At the community, they were encouraged to undertake 30 or more minutes of physical activity at moderate to vigorous intensity at least 5 days per week	Those randomized to CCR had greater knowledge than those in exercise-only CR or waitlist control. waitlist controls.

Quality of Life

A total of 16 studies reported outcomes on QoL [16,18-21,23-25,27-31,34-36]. Varying tools were used to measure change in QoL including the General Health Questionnaire (GHQ), Self-Rated Health (SRH), post-MI specific tool (MacNew QLMI), Minnesota Living with Heart Failure Questionnaire (MLHFQ), Hospital Anxiety and Depression Scale (HADS), Duke Activity status index (DASI), Self-rated health (visual analogue scale of EQ-5D-5L), 36-Item Short Form Survey (SF-36), 26-item Cardiac Depression Scale (CDS), MacNew QoL after Myocardial Infarction (QLMI), World Health Organization QoL (WHOQOL-BREF), Patient Health Questionnaire (PHQ-9) and Depression Anxiety Stress Scale (DASS-21). This makes it difficult to make comparisons across studies.

All the studies reported improvement in QoL of health and/or perception of health. Four studies reported data on anxiety and depression [20,25,30,35]. In three of the studies [20,30,35], there was no significant difference in anxiety and depression between the intervention and control groups while in one study [25], there was a significant reduction in depression (p=0.0). The QoL outcomes from the studies included are summarized in Table 4.

**Table 4:** Summary of health-related quality of life outcomes for the studies included. GeneralHealth Questionnaire (GHQ), Self-Rated Health (SRH), MacNew Heart Disease Quality of Life after Myocardial Infarction questionnaire (MacNew QLMI), Minnesota Living with Heart Failure Questionnaire (MLHFQ), Hospital Anxiety and Depression Scale (HADS), Duke Activity status index- (DASI), Self-rated health (SRH), (visual analogue scale of EQ-5D-5L), 36-Item Short Form Survey (SF-36), 26-item Cardiac Depression Scale (CDS), Mac New Quality of Life after Myocardial Infarction (QLMI), World Health Organization Quality of life (WHOQOL-BREF), Patient Health Questionnaire (PHQ-9), Depression Anxiety Stress Scale (DASS-21), Cardiac rehabilitation (CR), Chronic heart failure (CHF), Quality of life (QoL), Coronary artery disease (CAD), Yoga-based CR (Yoga-CaRe), EQ-5D-5L questionnaire (EQ-5D-5L), Integrated approach of Yoga Therapy (IAYT), Duke Activity Status Index (DASI), The World Health Organization Quality of Life Brief Version (WHOQOL-BREF), Cardiac Depression Scale (CDS), Nurse -led eHealth CR (NeCR), Heart failure (HF), Chronic heart failure (CHF), High-intensity aerobic interval training (HIAIT)

AUTHOR (YEAR)	CVD	Settings	INTERVENTION	QOL TOOL	QOL FINDINGS
Poortaghi et al., 2011 [16]	CAD	Home-based	Hospital-based CR followed by home visits by a community health nurse and nutritionist.	GHQ-28	Statistical difference in the general health (p=0.0001)
Xueyu et al., 2017 [18]	CHF	Hospital-based and home-based	Transitional care program - individual discharge plan prepared for management of CHF and low-intensity exercise (walking protocol).	MLHFQ	Significant improvement
Duan et al., 2018 [19]	CAD	Home-based	Web-based intervention - First 4 weeks of physical activity and subsequent 4 weeks of fruit and vegetable consumption	Hong Kong version of the World Health Organization Quality of Life questionnaire (brief version)	Improved QoL (p<0.001)
Peng et al., 2018 [20]	CHF	Home-based	Home-based Telehealth exercise training (brochure, exercise training, regular telephone and instant messaging follow-up and consultation)	MLHFQ, HADS	Significant improvement in QoL. No improvement in anxiety and depression
Ul-Haq et al., 2019 [21]	CAD	Hospital and home	Two phases: 1-2 weeks hospital and 6-7 weeks outpatient structured exercise program	GHQ, SRH, MacNew QLMI	All three measures confirmed better Health-related QoL than usual care (p<0.001)
Khalid et al., 2019 [23]	CAD	Hospital-based	Supervised resistance interval training in addition to Aerobic Interval Training	SF-36	Mean in the domains of energy/fatigue, emotional well-being and social functioning showed statistically significant differences (p = 0.013, 0.001 and 0.000)
Prabhakaran et al., 2020 [24]	CAD	Hospital-based	Yoga-based CR (Yoga-CaRe) - 13 direct contact sessions. The first 2 sessions were delivered individually and the rest in groups at the hospital.	EQ-5D-5L, Reintegration to Normal Living Index	Significant improvement in self-rated health and return to pre-activity
Sharma et al., 2020 [25]	CAD	Hospital and home	Three supervised integrated approach of Yoga Therapy (IAYT) classes at the hospital yoga centre supervised by a yoga therapist/cardiologist.	CDS, DASI	Significant reduction in depression (p=0.0), Significant increase in QoL (P=0.0)
Uddin et al., 2020 [27]	CAD	Home-based	In-hospital CR class, locally developed educational booklet, telephone follow up	WHOQOL-BREF	Significantly improvement in health-related QOL and overall perception of health
He et al., 2020 [28]	CAD	Hospital and home	Physician-supervised exercise followed by home-based exercise training (moderate continuous training)	SF-36	Improved physical-related quality of life
Arjunan et al., 2020 [29]	CHF	Hospital-based and home-based	Nurse-led CR - structured teaching, booklet on CR (Healthy way to the healthy heart) given at discharge and fortnightly phone reminders about good CR practices.	Health survey (SF-36) and MLHFQ	Significant improvement in physical component, mental component and disease-specific QoL.
Su et al., 2021 [30]	CAD	Home-based	NeCR (Nurse -led eHealth cardiac rehabilitation) - In-person session by the nurse to develop an individualised plan and after discharge, a health platform was used	Mac New, DASS-21	Improvement in health-related QoL (p<001). No significant difference in terms of anxiety, depression and stress.
Pakrad et al., 2021 [31]	CAD	Hospital-based and home-based	Hybrid CR - 1 month supervised and an additional 3 months remotely. Patients were given an app and communicated biweekly with the nurse for 1- 4 months	Health Survey (SF-36)	Significant improvement in the physical and mental component
Dolesky et al., 2018 [34]	HF	Hospitaland home	Moderate-intensity aerobic training (Interval training for 3 weeks, five days a week starting as an in-patient and continuing as an outpatient)	MLHFQ	Significant Improvement
Aranov et al., 2019 [35]	CAD	Hospital and home	Exercise in a controlled setting (medical centre) followed by home-based exercise	HADS, QoL assessed by a questionnaire developed by D.M. Aronov and V.P. Zaitcev	No significant difference in anxiety and depression, Improved QOL, significant improvement in QoL means score compared to baseline
Papathanasiou et al., 2020 [36]	CHF	Hospital-based	Modified group High-intensity aerobic interval training (HIAIT)	MLHFQ	Significant improvement in QoL (P<0.001)

Discussion

To our knowledge, this is the first systematic review to evaluate the effectiveness of CR in low-income and middle-income countries. This is a very important milestone, as it provides evidence that CR can be successfully implemented in low- and middle-income settings. The electronic search retrieved 1102 papers, of which 457 were assessed for eligibility. A total of 336 studies were excluded because they were conducted in HIC. Only 22 eligible studies were included in this study. This demonstrates that CR is still a new concept in many LMIC. In addition, the 22 studies retrieved were all conducted within the last decade (2011 - 2022), supporting the fact that CR is a recent addition to the CVD package of care in LMIC. The majority of the included studies were conducted in Asia, of which almost half were from China. We did not obtain any eligible full papers from Africa, pointing to a potential gap in the availability of CR services. This situation is likely to worsen in sub-Saharan Africa. The studies included in this review are summarized in Table 5.

**Table 5:** The main characteristics of the studies included in this review. High-intensity aerobic interval training (HIAIT), General Health Questionnaire (GHQ-28), Coronary artery bypass grafting (CABG), Percutaneous transluminal coronary angioplasty (PTCA), Cardiac rehabilitation (CR), Functional exercise capacity (FEC), Moderate intensity continuous training (MICT), Left ventricular ejection fraction (LVEF), 6MWD (6-minute walk distance), Peak oxygen uptake (VO2peak), m-Borg’s perceived exertion scale (mBPES), Quality of life (QoL), Cardiopulmonary exercise testing (CPX), Depression, anxiety and stress (DASS-21), Continuous care model (CCM), Nurse-led eHealth CR (NeCR), Coronary heart disease (CHD), Health-related quality of life (HRQoL), Myocardial infarction in the absence of obstructive CAD (MINOCA), Minnesota Living with Heart Failure Questionnaire (MLHFQ), Timed Up and go test (TUG test), Random blood sugar (RBS), systolic blood pressure (SBP), diastolic blood pressure (DBP), Graded cycle ergometer test (CE-test), Major adverse cardiovascular events (MACE)

Study	Country	Study Design	Sample	Target population	Setting	Intervention	Control	Duration	Outcomes
Poortaghi et al., 2011 [16]	Iran	RCT	80	Patients’ post-CABG, MI or PTCA	Home-based	Hospital-based CR with education and practical training in various rehabilitation measures and home visits by a community health nurse. Education regarding risk factors, nutrition, taking medication, and the necessity of continuing the program at home were given by the nurse and nutritionist. Practical training on measuring heart rate, detecting target heart rate, doing suitable exercises at home, setting the home exercise program, walking and jogging was performed by a team including a nurse and physiotherapist. The structure and the contents of the training course were handed out to the patients. At the end of the first and second months after the program, the researcher, as a community health nurse, visited twice at home.	Hospital-based CR only	2 months	The General Health Questionnaire (GHQ-28) was used to assess psychological and general health
Anari et al., 2015 [17]	Iran	Prospective interventional cohort study	108	Patients with a previous diagnosis of CAD (post CABG or PTCA)	Hospital-based	Exercise training program 3 times a week for 12 weeks, dietary recommendations based on BMI, WC and laboratory assessments. Comprehensive recommendations for diet and exercise.	None	12 weeks	CVD risk factors (BMI, WC, FBG, TG, HDL, SBP, DBP)
Xueyu et al., 2017 [18]	China	RCT	83	Stable Class II or III CHF (NYHA) with limitations in PA and comfortable at rest but symptomatic with or less than ordinary PA, 70 or more years	Hospital-based and home-based	Transitional care program (Nurse-led multi-disciplinary team, individual discharge plan prepared for management of CHF which included noting diet/drinking habits, monitoring weight, blood pressure and blood sugar, monitoring worsening of symptoms, medication use and daily PA instruction. Instructions were given orally and in written form) In addition, the intervention group participated in a low-intensity exercise-walking protocol. 5-10 min warm-up, 5-10 min relaxation. The intensity was 10 - 20% or a maximum 6MWD, target HR 5-10 bpm more than resting HR and Borg rating or perceived exertion 11 - 13.	Participated in the transitional care program but continued their usual daily physical activities and avoided high-intensity exercise	12 weeks	Minnesota Living with Heart Failure Questionnaire (MLHFQ), 6MWD Timed Up and Go (TUG) test
Duan et al., 2018 [19]	China	RCT	114	Outpatient with CAD	Home-based	Web-based intervention - First 4 weeks PA and subsequent 4 weeks on fruits and vegetable consumption, 2 web-based assessments. Patients were invited to access a Web-based health program via their home computer once a week during the 8 weeks.	Waiting control group	8 weeks	PA, FVC, healthy lifestyle (the synthesis of PA and FVC), internal resources (combination of intention, self-efficacy, and planning), and an external resource (social support) for PA and FVC behaviours, as well as perceived health outcomes (body mass index, quality of life, and depression).
Peng et al., 2018 [20]	China	RCT	98	CHF for at least 3 months, NYHA 1 to III, more than 18yrs, clinically stable with regular meds for at least 4 weeks	Home-based	Home-based Telehealth exercise training - 2 months intervention (printed brochure, exercise training education and 32 sessions exercise training, regular telephone or instant messaging follow-ups and consultations)	Usual care - Simple discharge education and regular follow-up visits at the clinic, no instruction regarding exercises	6 months	MLHFQ, 6MWD, resting heart rate (HR), Hospital Anxiety and Depression Scale and New York Heart Association (NYHA) classification.
Ul-Haq et al., 2019 [21]	Pakistan	RCT	206	Patients with the first MI	Hospital-based and home-based	Two phases: 1-2 weeks hospital and 6-7 weeks outpatient structured exercise program	Standard communication from the cardiologist and routine follow-up care (brief counselling regarding their health, medicine prescription and follow-up advice)	8 weeks	General Health Questionnaire (GHQ) and Self-Rated Health (SRH) and one post-MI specific tool (MacNew QLMI, lifestyle risk factors (smoking status, BMI)
Dorje et al., 2019 [22]	China	RCT	312	Patients 18 years and older with CAD post-PCI	Home-based	2-month intense program and 4-month step-down phase of smartphone-based CR and secondary prevention program delivered via social media platform WeChat (SMART-CR/SP).	Standard outpatient cardiology follow-up without formal CR and Secondary prevention.	12 months	Functional capacity (6MWD), BP, blood lipids, BMI, WHR, Generalized Anxiety Disorder 7 item scale and Patient Health Questionnaire 9-item scale, 12-item Short-Form Health Survey for quality of life. Behaviour change for smoking status (self-reported), PA (International PA questionnaire) and dietary habits (WHO Steps)
Khalid et al., 2019 [23]	Pakistan	RCT	26	Stable post-MI patients, 35 years and above	Hospital-based	Resistance interval training in addition to AIT. Duration was 35 - 40min. Intensity and resistance gradually increased to accommodate the exercise load. This supervised training protocol was followed thrice a week on alternate days for 6 weeks.	AIT on stationery cycle and treadmill interspersed with rest intervals. The session ended with a 10 - 15-minute cooldown	6 weeks	Primary outcomes - 6MWD and peak oxygen uptake (VO2), secondary outcome, Quality of Life
Prabhakaran et al., 2020 [24]	India	RCT	3959	Acute MI, 18-80 years, past 14 days	Hospital-based	Yoga-based CR (Yoga-CaRe) - 13 direct contact sessions spread over 12 weeks with the first session within 2 weeks of the index event. The first 2 sessions were delivered individually and the rest in groups at the hospital. Group sessions are 75 min with gentle yoga followed by a discussion on lifestyle and psychosocial concerns.	Enhanced standard care - 3 sessions of educational advice with the help of a leaflet, spread over the same period as the Yoga-Care sessions.	6 months	First occurrence of major adverse cardiovascular events (MACE) (composite of all-cause mortality, MI, stroke, or emergency cardiovascular hospitalization), self-rated health on the European Quality of Life–5 Dimensions–5 Level visual analogue scale at 12 weeks
Sharma et al., 2020 [25]	India	RCT	66	30 - 65 years old, recent MI on conservative management without involving conservative procedures (PCI, CABG), 10 days to 2 months post-MI, left ventricular dysfunction (NYHA class I and II, LVEF 30-50%.	Hospital-based and home-based	Three supervised integrated approach of Yoga Therapy (IAYT) classes 3 days per week for 12 weeks at the hospital yoga centre under the supervision of a yoga therapist/cardiologist. Patients were encouraged to continue at home on the other days and were given a written and digital guide	Standard care - pharmacological treatment and instructions from the cardiologist	12 weeks	Duke Activity Status Index (DASI) to measure quality of life, Metabolic equivalents, blood lipids (LDL, HDL, TG), Anxiety and depression assessed using the Hamilton Anxiety Rating Scale (HAM-A) and Cardiac Depression Scale (CDS)
Song et al., 2020 [26]	China	RCT	106	Patients with stable CHD	Home-based	Smart-phone-based telemonitored cardiac rehabilitation. CR involved an exercise prescription after cardiopulmonary exercise testing. Exercise was walking, 3-5 times a week for 30 min with 5-10 warm up and relaxation after the exercise	Routine follow-up	6 months	Changes in exercise tolerance - VO2 peak, difference in exercise habits, blood lipids, blood glucose
Uddin et al., 2020 [27]	Bangladesh	Quasi-RCT	142	Post-CABG	Home-based	CR group received an in-hospital CR class and was introduced to a locally developed educational booklet with details of a home-based exercise program then received monthly telephone calls for 12 months	Drug treatment and usual hospital follow-up	12 months	Coronary heart disease (CHD) risk factors, health-related quality of life (HRQOL), mental well-being, Maximal oxygen uptake
He et al., 2020 [28]	China	RCT	524	Patients with MINOCA	Hospital and Home-based	Exercise under the supervision of a physician in the hospital three times per week for 20–30 min on a treadmill or bicycle at 65%–75% of the symptom-limited maximal heart rate. Home-based exercise-training program three times a week (52 sessions/year) during the three years of moderate continuous training (MCT - cycle or run continuously on a treadmill at moderate intensity; 65%–75% of peak heart rate) for 37 minutes.	Usual care	3 years	All-cause mortality, MACE (cardiovascular death, nonfatal MI, HF, stroke and CV-related hospitalization
Arjunan et al., 2020 [29]	India	RCT	200	Patients admitted with CHF	Hospital-based and home-based	Nurse-led CR - structured teaching on the disease, diet, exercise, medication, home care, smoking cessation and lifestyle modification, on one 3 time at bedside, booklet on CR (Healthy way to healthy heart) given at discharge and fortnightly phone reminders about good CR practices.	Usual care - physician visits, nursing care, physiotherapy and the booklet	3 months	General and disease-specific quality of life, physiological measurements (BMI, BP and serum cholesterol)
Su et al., 2021 [30]	China	RCT	146	Initial diagnosis of CAD based on angiography or exacerbation of CAD in previously diagnosed	Home-based	NeCR (Nurse-led eHealth cardiac rehabilitation). In-person session by the nurse to develop an individualised plan for behaviour risk modification and orientation on the technology platform. After discharge, the eHealth platform helped patients gain knowledge of disease management and monitor goal attainment for health behaviour changes. The nurse provided feedback weekly on goal attainment and lifestyle modification in small groups through the WeChat platform (peer influence)	Usual care - 10 min didactic session on medication usage and lifestyle changes (PA, diet and smoking cessation) delivered by staff nurses of the study hospital	12 weeks	Lifestyle behaviours, psychological risk parameters and clinical outcomes
Pakrad et al., 2021 [31]	Iran	RCT	88	Post-CABG patients	Hospital-based and home-based	Hybrid CR - 1 month supervised an additional 3 months. remotely based on the continuous care model (CCM). Patients were given an app and communicated biweekly with nurses from months 1-4 to control risk factors	Traditional (1-month supervised)	4 months	Quality of life (QoL; SF-36; primary outcome), functional capacity (treadmill test), depression, anxiety and stress (DASS-21)
Cai et al., 2022 [32]	China	RCT	100	Patients with AF who underwent ablation	Home-based	Home-based patient-tailored and mobile application-guided cardiac telerehabilitation	12-week standard CR	12 weeks	improvement in Peak Vo2, adherence, physical activity, beliefs related to cardiovascular disease and exercise self-efficacy
Li et al., 2022 [33]	China	RCT	89	Patients 45 years and above were diagnosed with CAD by angiogram and received PCI	Hospital	Micro-lecture education to cover CR and secondary prevention. Micro-lectures were uploaded to the TV in the ward. Micro-lectures were demonstrated twice a day (10 am and 1600) for 5-8min.	Educational brochures and standard didactic CR education on diagnosis and treatment of CAD, self-management of risk factors, medication, nutrition and exercises.	12 weeks	Secondary outcomes of smoking status, exercise status, and 6MWD
Doletsky et al., 2018 [34]	Russia	RCT	46	Decompensated HF (CHF with moderate or severe symptoms - class III or IV NYHA)	Hospital-based and home-based	Moderate intensity aerobic training. Interval training for 3 weeks, five days a week starting as an in-patient and continuing as an outpatient after discharge (15 sessions/patient). All patients were followed up with weekly calls and monthly visits to the hospital	Physical activity recommendations are given at discharge	3 months	6MWD, cardiopulmonary exercise testing (CPX), MLHFQ
Aranov et al., 2019 [35]	Russia	RCT	36	Male patients 3-8 weeks after CABG	Medical centre and home-based	60 min exercise in a controlled setting (medical centre) 3 times a week for 4 months followed by home-based exercise	Recommendation to perform exercise at home in an uncontrolled setting	12 months	CVD risk factors (BMI, SBP, DBP, blood lipids, blood glucose), physical activity, dietary habits, clinical outcomes, quality of life
Papathanasiou et al., 2020 [36]	Bulgaria	RCT	120	Stable CHF, NYHA classes II to IB, LVEF equal or more than 40, clinically and pharmacologically stable for more than 3 months	Hospital-based	Modified group High-intensity aerobic interval training (HIAIT)	Moderate intensity continuous training (MICT)	12 weeks	Functional exercise capacity (FEC), assessed with a six-minute walk test, and peak oxygen uptake (VO2peak), m-Borg’s perceived exertion scale (mBPES), and quality of life (QoL)
Chaves et al., 2019 [37]	Brazil	RCT	115	Patients post- CABG, myocardial infarction (MI) or percutaneous transluminal coronary angioplasty (PTCA)	Hospital-based and home-based	There was comprehensive CR (CCR) - exercise and education and CR (exercise only). The exercise comprised 36, 1 hour supervised sessions. Individualized exercise prescription based on stress test. At the community, they were encouraged to undertake 30 or more minutes of PA at moderate to vigorous intensity on at least 5 days per week as recommended by practice guidelines. In CCR, 24 education sessions 30 minutes weekly in groups, they received an education workbook	Usual care - patients follow-up appointments with physician (no CR due to lack of capacity)	12 months	Functional capacity (incremental shuttle walk test, 7 metabolic equivalents target), BP at rest, blood lipids, BMI, WC, depressive symptoms (Patient Health Questionnaire-9), CVD knowledge, heart-healthy behaviours (exercise, diet and smoking), mortality (all-cause and cardiovascular), morbidity

CR Models in LMIC

This study revealed a variety of models that are effective for CR delivery in LMIC. The majority of studies have incorporated home-based/community components [16-22,25,27,32,34,35,37], a strategy that has been proposed to improve the utilization of CR in LMIC [6]. Home-based programs are facilitated through task shifting to other cadres, such as nurses and nutritionists, and the use of technology to facilitate remote monitoring and communication. The use of mobile-based and home-based exercise programs is another strategy proposed to improve uptake and retention in CR programs [2]. Seven studies [19,20,22,26,30,31,32] implemented interventions that involved the use of technology, such as mobile phones or computers. One limitation of the technology-based model is the exclusion of patients who are not technology-savvy. We also need to acknowledge that a significant proportion of patients with CVDs may be elderly and, therefore, may be unable to use this technology. It is important that alternative options are made available for patients who are unable to use these new technologies.

Target Population

Most studies have targeted patients with CAD. It is important to note that the American Heart Association (AHA) and American College of Cardiology (ACC) give a Class 1 recommendation for referral to CR services for patients with myocardial infarction, coronary artery bypass graft, percutaneous coronary intervention, chronic stable angina, heart failure, peripheral arterial disease, and CVD prevention in women [1]. It is therefore important to expand the scope of this service to include other CVDs, especially patients with heart failure, a condition common in LMIC. The studies included in this review that targeted patients with CHF [18,20,29,34,36] demonstrated that CR can lead to improvements in functional capacity, risk factor management (BP, blood glucose, blood lipids), and QoL.

It is also worth noting that all CR models used in the studies differed in terms of staff, duration, type of exercise, settings, and materials used. Despite this, the benefits of CR, especially in the improvement of functional capacity, QoL, and clinical outcomes, are evident. It is therefore important to realize that it may not be realistic to develop a standard CR model that can be utilized across all LMICs. Service providers must use the resources and expertise available and consider patient preferences as well as the cultural setting when designing CR programs. A good example can be drawn from studies conducted in India [24,25]. The practice of yoga is believed to have originated in India; therefore, it is not surprising that the study investigators considered that it would be an acceptable form of exercise to incorporate into CR programs. The intervention by Sharma et al. [25] involved an integrated approach involving Yoga (IAYT) classes supervised by a yoga therapist or cardiologist. This intervention resulted in improved functional capacity, reduced depression, and an improved QoL. The intervention by Prabhakaran et al., which involved 13 yoga sessions delivered initially individually and subsequently in groups, also resulted in significant improvement in self-rated health and return to pre-activity.

Effectiveness of CR in LMIC

This study demonstrated that CR was effective in the treatment of LMIC. Unfortunately, all studies included in this review were from middle-income countries. We recognize that low-income countries may have additional resource constraints and other competing health priorities that may pose additional challenges to the expansion of CR services. All studies that reported functional capacity and QoL outcomes demonstrated that CR was effective regardless of the delivery model used. Studies assessing risk factor reduction (behavioral and physiological) have shown mixed results. It is worth conducting further assessments of these studies to determine what could have resulted in the mixed results.

Study limitations

There are few limitations to our study. The number of published studies from LMIC is limited and it may not accurately reflect the real situation. We did not include studies from HIC which may provide different results compared to the studies from LMIC. Finally, despite the best of our efforts to minimize it, there is always a chance of selection bias in these studies.

## Conclusions

CR in LMIC has clear benefits, especially in improving the functional capacity and QoL. CVD in Sub-Saharan Africa tends to affect a younger population and therefore these services are critical to ensure these individuals recuperate and continue to be productive members of society. CR practitioners in LMIC will have to utilize available resources (staff, technology, materials, practices) to design and deliver these programs. In addition, they have to take into consideration the cultural practices and patient preferences to improve uptake. It is clear that regardless of the model used, patients receiving these services will benefit more than patients receiving no CR services at all.
